# Efficacy and Safety of Antibody-Drug Conjugates for Lung Cancer Therapy: A Systematic Review of Randomized and Non-Randomized Clinical Trials

**DOI:** 10.3390/pharmaceutics17050608

**Published:** 2025-05-03

**Authors:** Matteo Gallina, Anna Carollo, Anna Gallina, Sofia Cutaia, Sergio Rizzo, Alessio Provenzani

**Affiliations:** 1 Department of Biological, Chemical and Pharmaceutical Sciences and Technologies (STEBICEF), School of Specialization in Hospital Pharmacy, University of Palermo, Viale delle Scienze, Ed.16-17, 90128 Palermo, Italy; matteo.gallina01@you.unipa.it; 2 Clinical Pharmacy Service, Mediterranean Institute for Transplantation and Advanced Specialized Therapies (IRCCS ISMETT), Via Ernesto Tricomi, 5, 90127 Palermo, Italy; 3 Department of Chemical, Biological, Pharmaceutical, and Environmental Sciences, School of Specialization in Hospital Pharmacy, University of Messina, Viale Ferdinando Stagno d’Alcontres, 31, 98166 Messina, Italy; agallina@ismett.edu; 4 Medical Oncology Service, Mediterranean Institute for Transplantation and Advanced Specialized Therapies (IRCCS ISMETT), Via Ernesto Tricomi, 5, 90127 Palermo, Italy; scutaia@ismett.edu (S.C.); srizzo@ismett.edu (S.R.)

**Keywords:** non-small cell lung cancer, antibody-drug conjugates, targeted therapy, tumor-agnostic therapy, systematic review

## Abstract

**Background/Objectives**: Lung cancer is the leading cause of cancer-related deaths worldwide. Non-Small-Cell Lung Cancer (NSCLC) accounts for 80–90% of all lung cancers. Antibody-Drug Conjugates (ADCs) represent an expanding targeted therapy option for the treatment of NSCLC. The aim is to perform a systematic literature review to evaluate the efficacy and safety profiles of ADCs currently undergoing clinical trials for the treatment of NSCLC. **Methods**: The study adhered to the Preferred Reporting Items for Systematic reviews and Meta-Analyses (PRISMA) statement. Literature searches were conducted in PubMed, ClinicalTrial.gov and Web of Science databases, covering the period from 2014 to 2024. Only randomized and non-randomized phase II-IV clinical trials focusing on ADC-based therapies for adult patients affected by NSCLC were selected. The Revised Cochrane Risk-of-Bias Tool for Randomized Trials (RoB 2.0) and the Risk of Bias in Non-randomized Studies of Interventions (ROBINS-I) were used to evaluate the overall risk of bias in the included randomized and non-randomized studies, respectively. While GRADE (Grading of Recommendations, Assessment, Development and Evaluations) methodology was used to assess the certainty of the evidence. Efficacy endpoints were categorized based on primary outcomes while safety was assessed through the frequency and severity of Treatment-Emergent Adverse Events (TEAEs), and a qualitative summary of the findings was conducted. **Results**: A total of seven studies, including three randomized, three non-randomized, and one without specific allocation, were included, comprising 1287 patients, with 693 (54%) men, and an average age of 63 years old. Two studies were deemed to have a low risk of bias, while six had a moderate risk or some concerns. Five ADCs were evaluated: trastuzumab deruxtecan (T-DXd), trastuzumab emtansine (T-DM1), telisotuzumab vedotin, patritumab deruxtecan, and datopotamab deruxtecan (Dato-DXd). T-DXd demonstrated superior efficacy in HER2-overexpressing and HER2-mutant NSCLC, with an ORR of 52.9% and 49.0%, respectively. However, HER2-mutant patients exhibited a longer median DOR (16.8 vs. 6.2 months) but a higher incidence of grade ≥ 3 TEAEs (38.6% vs. 22%). T-DM1 showed modest efficacy, with an ORR of 20% in HER2-overexpressing NSCLC and 6.7% in HER2-mutant patients. Dato-DXd demonstrated improved ORR (26.4% vs. 12.8%) and PFS (4.4 vs. 3.7 months) compared to docetaxel. Patritumab deruxtecan achieved an ORR of 39% in EGFR-mutant NSCLC, while telisotuzumab vedotin exhibited limited activity in c-MET-positive NSCLC (ORR 9%, median DOR 7.5 months). Frequency and severity of TEAEs varied across ADCs, with ILD being a major concern, highlighting the need for strict patient monitoring and early intervention to mitigate severe adverse events. **Conclusions**: ADCs represent a promising advancement in NSCLC treatment, offering targeted therapeutic options beyond conventional chemotherapy and immunotherapy. T-DXd has emerged as the most effective ADC for HER2-mutant NSCLC with manageable safety profile, whereas Dato-DXd provides a viable alternative for TROP2-expressing tumors. While ADCs offer significant clinical benefits, careful patient selection and proactive management of adverse events remain crucial. Ongoing and future trials will further refine the role of ADCs in personalized NSCLC treatment, potentially expanding their tumor-agnostic use to broader patient populations.

## 1. Introduction

Lung cancer is the second most common cancer in the U.S. and the leading cause of cancer-related deaths. Non-Small-Cell Lung Cancer (NSCLC) represents 80–90% of cases, with approximately 70% non-squamous and 30% squamous histology [1]. It accounts for approximately one in five cancer fatalities annually, surpassing colon, breast, and prostate cancer deaths combined [2]. The American Cancer Society projects 226,650 new cases and 124,730 deaths in 2025 with men and women affected nearly equally [3]. In Europe, nearly 500,000 cases were diagnosed in 2022 [4,5]. The lungs are also the most common site of metastasis, affecting over 50% of patients with secondary tumors from breast, colorectal, and renal cancers [6,7].

NSCLC treatment is closely aligned with disease stage [8]. Early-stage NSCLC (IA–IIB) is primarily managed with surgical resection, often followed by adjuvant chemotherapy or radiotherapy based on tumor size and lymph node involvement. Locally advanced NSCLC (stages IIIA–IIIB) typically requires multimodal therapy, including chemoradiotherapy, with surgery considered for resectable cases. Unresectable tumors may be treated with immunotherapy (e.g., PD-L1 inhibitors like durvalumab or atezolizumab) post-chemoradiotherapy [1,4]. In metastatic NSCLC (stage IV), systemic therapies, including chemotherapy, immunotherapy, and targeted treatments, are guided by molecular profiling to identify actionable mutations such as Epidermal Growth Factor Receptor (EGFR) and Anaplastic Lymphoma Kinase (ALK) rearrangements or high PD-L1 expression [9,10]. Despite advances in immunotherapy and targeted therapies, disease progression often necessitates chemotherapy, which remains limited in efficacy with significant side effects [11,12].

Antibody-Drug Conjugates (ADCs) are an expanding targeted therapy for the treatment of NSCLC, leveraging monoclonal antibodies to deliver cytotoxic molecules selectively into tumor cells [13]. The antibody-drug linkage ensures release only upon ADC binding or internalization, enhancing efficacy and minimizing off-target effects [14]. First conceptualized by Paul Ehrlich in 1913, ADCs initially faced challenges like unstable linkers and low cytotoxic payloads which resulted in minimal tumor targeting [15]. Nevertheless, advances in antibody engineering and linker technology have significantly improved stability and efficacy [16]. Key factors in ADC development include selecting optimal cancer targets, high-quality antibodies, and stable linkers that release the toxin selectively inside the tumor cells. Innovations like PEG linkers enhance solubility, reduce aggregation, and decrease immunogenicity, ultimately improving ADC performance [17].

Precision oncology has further revolutionized treatment with tumor-agnostic therapies targeting specific genetic mutations or molecular alterations driving tumor progression, regardless of tumor origin [18]. In NSCLC, these therapies have shown significant promise in patients with actionable mutations like EGFR, ALK, c-ros oncogene 1 (ROS1), Mesenchymal–Epithelial Transition Factor (c-MET), and Trophoblast cell surface antigen 2 (TROP2), enabling a personalized treatment approach beyond traditional histology, leading to expanding therapeutic use to broader patient populations [19].

The launch of gemtuzumab ozogamicin in 2000 for CD33-positive acute myeloid leukemia (AML) marked the beginning of the ADC development [20]. While no new ADCs reached the market during the decade following its withdrawal in 2010, a surge of development has since transformed the field [21]. ADCs now target a variety of hematologic and solid tumors, including NSCLC, with many candidates in advanced phases of clinical trials [13,14,22]. Trastuzumab deruxtecan (T-DXd) is currently the first and only ADC approved by both the Food and Drug Administration (FDA) and the European Medicines Agency (EMA) for treating adult patients with advanced NSCLC harboring an activating HER2 (ERBB2) mutation [23,24]. In April 2024, the FDA granted accelerated approval for T-DXd in adult patients with unresectable or metastatic HER2-positive (IHC3+) solid tumors, including NSCLC, who have received prior systemic therapy and lack satisfactory alternatives. This tumor-agnostic approval broadens patient eligibility and clinical utility. With several ADCs in late-stage development, treatment paradigms continue to evolve, driving innovation and market growth [22,25].

The primary aim of this study is to conduct a systematic literature review to evaluate the efficacy and safety profiles of ADCs currently undergoing clinical trials for the treatment of NSCLC. By employing a meticulously designed and highly selective search strategy, the study focuses on the latest clinical research exploring ADCs targeting specific mutations or overexpressed markers in patients affected by NSCLC. Through a comprehensive analysis, this manuscript seeks to provide evidence-based insights that empower healthcare professionals and policymakers to incorporate innovative, safe, and effective therapies into clinical practice, thereby improving patient outcomes.

## 2. Materials and Methods

### 2.1. Study Design and Search Strategy

The systematic review was performed in accordance with the Preferred Reporting Items for Systematic reviews and Meta-Analyses (PRISMA) statement [26], ensuring both transparency and reproducibility. The search strategy used a combination of Medical Subject Headings (MeSH) terms and Boolean operators, based on existing literature, associated with non-small cell lung cancer and ADC-based therapy in adult patients. The literature research was conducted using the following electronic databases to identify relevant records to include in the study: PubMed, ClinicalTrial.gov and Web of Science. The same search strategy was consistently applied across all databases. The last literature search was conducted on 30 September 2024.

### 2.2. Inclusion and Exclusion Criteria

To create a structured and comprehensive review, the inclusion and exclusion criteria were carefully developed using the PICOS framework (Population, Intervention, Comparison, Outcome, and Study Design). The search strategy was meticulously designed to capture only studies directly addressing the efficacy and safety profiles of ADCs in the treatment of NSCLC. Eligible studies focused on adult patients diagnosed with NSCLC at any stage of the disease, from early-stage (Ia) to advanced (IV). These studies examined treatment regimens where ADCs were used either alone or in combination with chemotherapy or immunotherapy. For comparison, the control groups included patients receiving either chemotherapy or immunotherapy without ADCs. The efficacy and safety outcomes assessed are those evaluated in clinical studies for experimental oncology drugs. Specifically, the efficacy outcomes included the confirmed objective response rate (ORR), defined as the sum of the confirmed complete response (CR) rate and partial response (PR) rate; the duration of response (DOR), defined as the time from date of initial response to the date of disease progression or death due to any cause for patients with a confirmed CR or PR; the progression-free survival (PFS), defined as the time from the date of random assignment to the earliest date of the first objective documentation of radiographic disease progression or death due to any cause; the overall survival (OS), defined as the time from the date of random assignment to the date of death due to any cause; the disease control rate (DCR), related to ORR, that describes the percentage of patients with advanced cancer whose therapeutic intervention has led to a CR, PR, or stable disease [27].

The safety of the ADCs included in this systematic review was assessed based on the frequency and severity of treatment-emergent adverse events (TEAEs), classified according to the Common Terminology Criteria for Adverse Events (CTCAE), version 5.0, where explicitly stated [28]. Only randomized and non-randomized phase II to IV clinical trials were included, provided they were published in English within the last decade. The focus was on high-quality research that specifically addressed ADC-based therapies for adult NSCLC patients. Studies were excluded if they involved patients with cancers other than NSCLC or those who did not receive ADC-based treatments. Research focusing on pediatric populations was not considered relevant to this review. Additionally, studies that lacked full-text availability or had not yet published their results were excluded. Non-peer-reviewed manuscripts, preclinical investigations, reviews, case reports, and phase I or I/II clinical trials—such as dose-finding or proof-of-concept studies—were also deemed ineligible. Observational studies, cost-effectiveness analyses, editorials, expert opinions, conference proceedings, and expanded-access programs were similarly excluded. Finally, studies not available in English or published before 2014 were omitted to ensure a consistent and recent evidence base. By applying these rigorous criteria, the review ensured a focused and high-quality synthesis of the literature on ADC therapies for NSCLC.

### 2.3. Selection of Studies, Data Extraxtion and Analysis

The study selection process adhered to the PRISMA flow diagram. Before starting the record screening process, duplicates were semi-automatically removed using the free version of the Rayyan^®^ tool. Afterwards, the overall articles were subjected to the screening by titles and abstracts to eliminate irrelevant papers according to article type and publication date. Full-text copies of the retrieved articles were assessed for eligibility to determine whether they contained relevant information for the systematic review according to the chosen inclusion criteria. Results from each study were categorized based on primary outcomes, and a qualitative summary of the findings was conducted. Subgroup analysis comparing HER2-mutant and HER2-overexpressing NSCLC populations treated with trastuzumab deruxtecan was carried out. Two reviewers independently screened and assessed the articles, resolving any issues through discussion, while a third author reviewed the overall process.

### 2.4. Quality Assessment of the Included Studies

The global risk of bias assessment of the included RCTs was carried out using the Revised Cochrane Risk-of-Bias tool for randomized trials (RoB 2.0), version of 22 August 2019 [29]. It is structured into five domains that refer to the randomization process, deviations from intended interventions, missing outcome data, measurement of the outcome, and selection of the reported results. A study is defined as having a low risk of bias when all domains are adequately met, at risk of unclear bias, with some concerns, when quality criteria are not reported or are unclear, and having a high risk of bias when one or more of the domains are not adequately met. While the Risk of Bias in Non-randomized Studies of Interventions (ROBINS-I) [30] tool was used to assess the quality of non-randomized trials included in the systematic review, comparing them to an ideal randomized trial. It assesses seven domains: confounding bias, which arises from failure to control for external variables; selection bias, related to how participants are chosen; misclassification of interventions; deviations from intended interventions; measurement bias in outcome assessment; bias due to missing data; and selective reporting of results. Each domain is judged as having low, moderate, serious, or critical risk of bias. A comprehensive overall risk of bias judgment combines these assessments to provide insight into the study’s reliability. The GRADE (Grading of Recommendations, Assessment, Development and Evaluations) methodology was used to assess the certainty of the evidence for the included studies to evaluate the reliability of the outcomes reported in the systematic review [31]. The analysis considered four main aspects: the risk of bias, heterogeneity, accuracy and indirectness. Then, GRADE classifies evidence into high, moderate, low and very low certainty [32].

### 2.5. Registration and Protocol

The reviews protocol has been registered on PROSPERO 2025 CRD42025643511. Available from: https://www.crd.york.ac.uk/prospero/display_record.php?ID=CRD42025643511 (accessed on 5 March 2025).

## 3. Results

### 3.1. Literature Search Results and Quality Assessment

**Figure 1:**
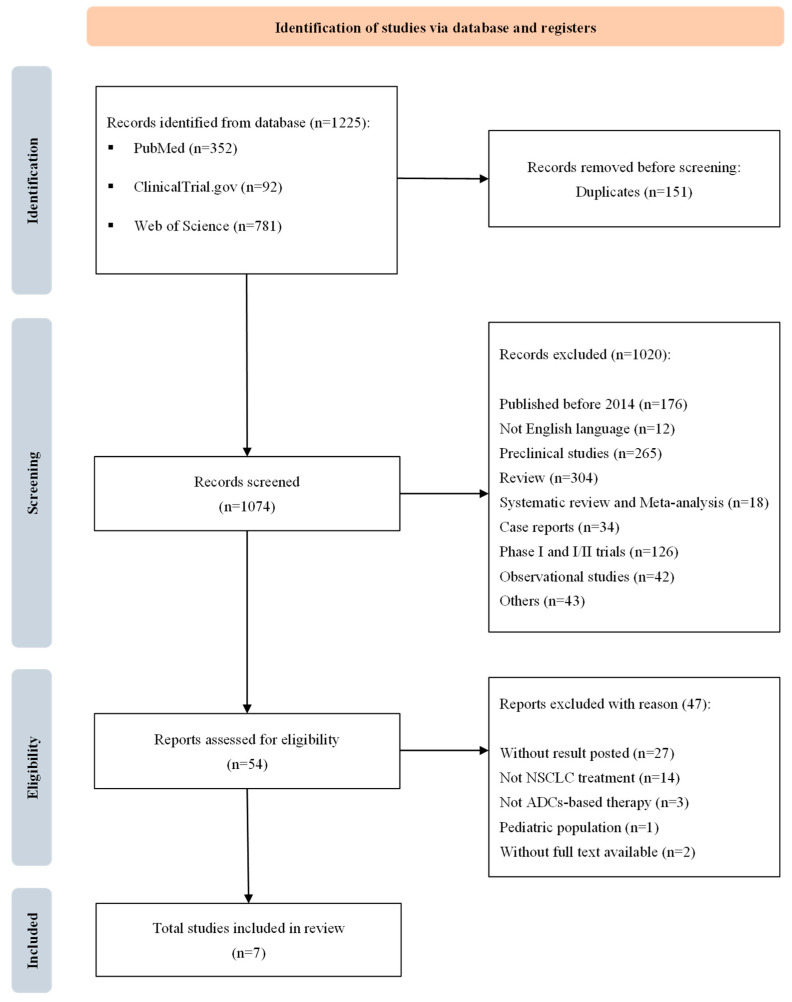
PRISMA flow chart of database search and article screening in the study.

The PRISMA flowchart of the search strategy and study selection process is shown in Figure 1. The systematic search yielded 1225 records: 352 from PubMed, 781 from Web of Science, and 92 from ClinicalTrials.gov. After removing 151 duplicates, 1074 records were screened, and 1020 were excluded for various reasons: 176 were published before 2014; 265 were preclinical studies; 304 were reviews; 126 were phase I or I/II trials; 42 were observational studies; 34 were case reports; 18 were systematic reviews or meta-analyses; 12 were in non-English languages; and 43 were excluded for other reasons (e.g., cost-effectiveness analyses, letters, expert opinions, conference proceedings, or expanded-access programs). After full-text screening of 54 records for eligibility based on the PICOS criteria, 47 were excluded: 27 lacked results; 14 involved the wrong population (not NSCLC); 3 involved the wrong intervention (non-ADC therapies); 1 involved a pediatric population; and 2 were incomplete studies. Ultimately, seven clinical trials met the inclusion criteria and were included in review for further considerations. 

**Figure 2:**
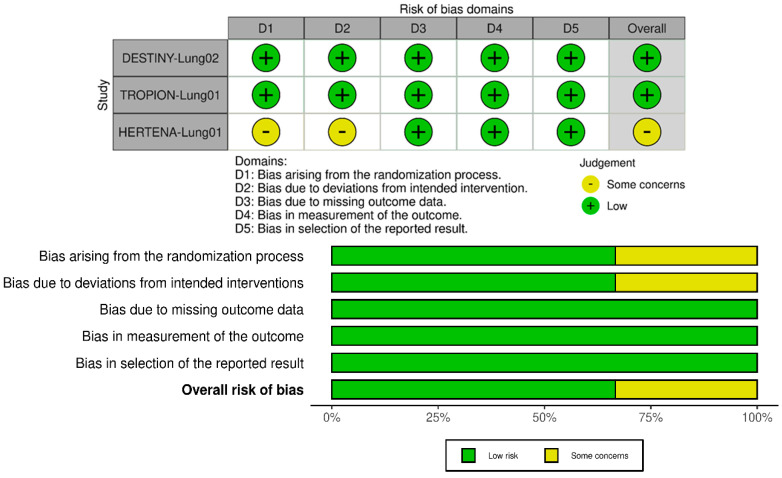
Risk of bias traffic light (**above**) and summary (**below**) plots of Revised Cochrane risk-of-bias tool for randomized trials (RoB 2.0) assessments for the randomized controlled trials (RCTs) included in the review. Each domain is judged as having low, some concerns or high risk of bias.

The visual representations of the risk of bias assessment for the RCTs included in the review are shown in Figure 2. The DESTINY-Lung02 [33] study demonstrates an overall low risk of bias across all domains, supported by robust randomization, effective blinding, objective outcome measurement criteria, and thorough data management, ensuring reliable results. The TROPION-Lung01 [34] study demonstrates a robust methodology with minimal risk of bias across all domains. Randomization was effectively implemented, and blinding of outcome assessment mitigated potential performance bias inherent in open-label designs. Furthermore, the trial adhered to its pre-registered protocol, ensuring transparency in outcome reporting. Overall, the study’s design and execution provide high confidence in the validity of its findings. In contrast, the HERTENA-Lung01 [35] study raises some concerns regarding bias in the randomization and intervention adherence domains, primarily due to its open-label design and limited details on the randomization process. However, the use of a blind independent central review (BICR) based on RECIST v1.1 for outcome assessment mitigates the potential impact of bias.

**Figure 3:**
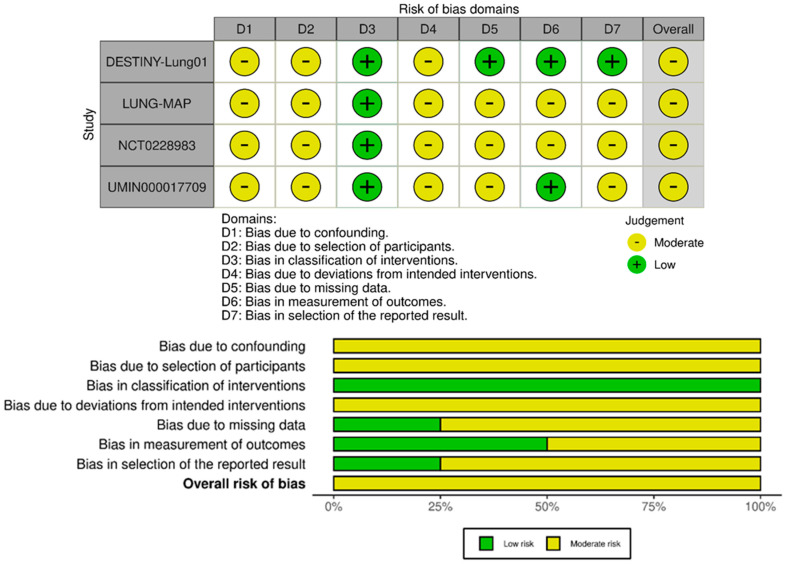
Risk of bias traffic light (**above**) and summary (**below**) plots of Risk of Bias in Non-randomized Studies of Interventions (ROBINS-I) assessments for the non-randomized clinical trials included in the review. Each domain is judged as having low, moderate, serious, or critical risk of bias.

The visual representations of the risk of bias assessment for the non-randomized clinical trials included in the review are shown in Figure 3. All studies showed an overall moderate risk of bias. Specifically, the DESTINY-Lung01 [36] study has an overall moderate risk of bias, mainly due to the lack of randomization and the open-label design, which may introduce unmeasured confounding and unintended bias. However, rigorous outcome monitoring and the use of BICR significantly mitigate the risk. The UMIN000017709 [37] study has some methodological limitations, especially in the domains related to confounding and participant selection. Nonetheless, the intervention follows a standardized approach and adheres to well-established protocols, with no evidence of systematic errors in classification. Additionally, the results reported no missing data, ensuring the integrity of the dataset, while the limited sample size narrows the margin for error. The LUNG-MAP [38] and NCT0228983 [39] studies demonstrate comparable moderate overall bias, primarily due to their single-arm designs, lack of randomization, and limited control for confounders. Despite these limitations, both studies excel in their systematic approach to intervention delivery and outcome evaluation. Particularly, the LUNG-MAP study benefits from clear intervention classification and predefined outcome measures, while NCT0228983 is strengthened by rigorous eligibility criteria for HER2 positivity and adherence to RECIST v1.1 for objective outcome assessment.

### 3.2. Baseline Characteristics of the Included Studies

The baseline characteristics of the seven included clinical trials are shown in Table 1. Three studies are randomized (DESTINY-Lung02; TROPION-Lung01; HERTENA-Lung01), three are non-randomized (DESTINY-Lung01; NCT0228983; UMIN000017709), and one study is without specific allocation (LUNG-MAP SUB-STUDY). All the selected studies completed phase II clinical trials, and one study is an active phase III trial. The total number of participants within the studies was 1287, with 693 (54%) men, and an average age of 63 years old. The year of each clinical study refers to the last update posted on ClinicalTrial.gov at the time of the literature search.

The overall characteristics of the ADCs used within the included clinical trials are shown in Table 2. A total of five ADCs targeting four different tumor-related markers were included in the review. Specifically, trastuzumab deruxtecan and trastuzumab emtansine targeting HER2; datopotamab deruxtecan targeting TROP2; patritumab deruxtecan targeting HER3; telisotuzumab vedotin targeting c-MET. Among these, three ADCs are currently approved by the FDA for the treatment of breast cancer, including trastuzumab deruxtecan, trastuzumab emtansine and datopotamab deruxtecan. The first two ADCs have also been approved in Europe by the EMA for the same therapeutic indication. Moreover, trastuzumab deruxtecan has been approved by both the FDA and EMA for the treatment of adult patients with advanced HER2-positive NSCLC and gastric cancer.

### 3.3. ADC Targeting HER2

Two trastuzumab-based ADCs have been designed to target HER2, a key oncogenic driver in a subset of NSCLC, combining a monoclonal antibody directed against HER2 with two different cytotoxic payloads, enabling selective delivery of chemotherapy to HER2-positive tumor cells while minimizing off-target effects. The primary efficacy and safety outcomes assessed in the included clinical trials are summarized in Table 3.

#### 3.3.1. Trastuzumab Deruxtecan (T-Dxd)

The efficacy and safety profiles of trastuzumab deruxtecan (T-DXd) for lung cancer treatment has been recently evaluated in two different clinical trials: DESTINY-Lung02, published in September 2023, and DESTINY-Lung01, published in April 2024. In the multicenter DESTINY-Lung02 trial, T-DXd was evaluated at two doses, 5.4 mg/kg and 6.4 mg/kg, in 152 patients with HER2-mutated metastatic NSCLC [33]. For the 5.4 mg/kg cohort, the confirmed ORR was 49.0% (95% CI: 39.0–59.1), with a median DOR of 16.8 months (95% CI: 6.4–not estimable [NE]). The median PFS was 9.9 months (95% CI: 7.4–NE), and the median OS was 19.5 months (95% CI: 13.6–NE). While for the 6.4 mg/kg cohort, the confirmed ORR was higher, at 56.0% (95% CI: 41.3–70.0), with a NE median DOR (95% CI: 8.3–NE). The median PFS and OS were 15.4 months (95% CI: 8.3–NE) and NE (95% CI: 12.1–NE), respectively. Regarding safety, grade ≥ 3 TEAEs occurred in 38.6% of patients receiving 5.4 mg/kg and 58.0% of those receiving 6.4 mg/kg. Adjudicated interstitial lung disease (ILD) was adjudicated in 12.9% of the 5.4 mg/kg cohort (grade ≥ 3: 2.0%; grade 5: 1.0%) and 28.0% of the 6.4 mg/kg cohort (grade ≥ 3: 2.0%; grade 5: 2.0%). Common TEAEs included nausea, 67.3% for 5.4 mg/kg and 82.0% for 6.4 mg/kg, neutropenia, 42.6% and 56.0%, fatigue, 44.6% and 50.0%, and decreased appetite, 39.6% and 50.0%, respectively.

In the multicenter DESTINY-Lung01 study, T-DXd was evaluated in 181 patients with HER2-overexpressing NSCLC at 6.4 mg/kg in Cohort 1 and 5.4 mg/kg in Cohort 1A [36]. In Cohort 1, the ORR was 26.5% (95% CI: 15.0–41.1), with a median DOR of 5.8 months (95% CI: 4.3–NE). The median PFS was 5.7 months (95% CI: 2.8–7.2), while the median OS was 12.4 months (95% CI: 7.8–17.2). In Cohort 1A, the ORR was higher, at 34.1% (95% CI: 20.1–50.6), with a median DOR of 6.2 months (95% CI: 4.2–9.8). The median PFS and OS were 6.7 months (95% CI: 4.2–8.4) and 11.2 months (95% CI: 8.4–NE), respectively. Safety data showed grade ≥ 3 TEAEs in 53% of patients in Cohort 1 and 22% in Cohort 1A. Adjudicated ILD or pneumonitis occurred in 20% of patients in Cohort 1 (grade 1/2: 14%; grade 5: 6%) and 5% in Cohort 1A (grade 2: 2%; grade 5: 2%). Common TEAEs included nausea (59% in Cohort 1, 73% in Cohort 1A), fatigue (59% and 71%), and decreased appetite (45% and 46%).

#### 3.3.2. HER2-Mutant Versus HER2-Overexpressing NSCLC Populations

Subgroup analysis comparing HER2-mutant and HER2-overexpressing NSCLC populations treated with T-DXd revealed distinct efficacy and safety profiles. Particularly, in HER2-mutant patients, T-DXd achieved a higher DOR of 16.8 months (95% CI: 6.4–NE) at a dose of 5.4 mg/kg, compared to a DOR of 6.2 months (95% CI: 4.0–11.7) in HER2-overexpressing patients. However, ORR were comparable, at 49.0% (95% CI: 39.0–59.1) and 52.9% (95% CI: 27.8–77.0) for HER2-mutant and HER2-overexpressing groups, respectively. Nevertheless, safety profiles differed, with Grade ≥ 3 TEAEs occurring in 38.6% of HER2-mutant patients versus 22% of HER2-overexpressing patients. These findings suggest that while both populations benefit from T-DXd, HER2-mutant patients experience longer response durations but a higher incidence of serious adverse events.

#### 3.3.3. Trastuzumab Emtansine (T-DM1)

The efficacy and safety profiles of trastuzumab emtansine (T-DM1) for lung cancer treatment has been recently evaluated in two different clinical trials: UMIN000017709 (2018) and NCT0228983 (2019). The 2018 study focused on patients with HER2-mutated NSCLC, characterized by activating HER2 mutations such as exon 20 insertions, representing a rare molecular subtype (1–3% of NSCLC cases) [37]. In phase II, single-arm trial of T-DM1, 3.6 mg/kg once every three weeks, in 15 patients with HER2-positive NSCLC, limited efficacy was observed. Particularly, the ORR was 6.7% (90% CI: 0.2–32.0), with only one patient achieving a partial response. The median PFS was 2.0 months (90% CI: 1.2–4.0), while the median OS was 10.9 months (90% CI: 4.4–12.0). Safety analysis revealed grade ≥ 3 TEAEs such as thrombocytopenia (40%) and hepatotoxicity (20%), with no treatment-related deaths. These findings indicate that T-DM1 has limited activity in this patient population.

In contrast, the 2019 study targeted patients with HER2-overexpressing NSCLC, defined by immunohistochemistry (IHC) 2+ or 3+, regardless of HER2 mutation status [39]. In this phase II study evaluating T-DM1, 3.6 mg/kg once every three weeks, in 49 patients with HER2-overexpressing NSCLC, efficacy varied between HER2 IHC 2+ (Cohort 1) and IHC 3+ (Cohort 2). In the Cohort 1, no objective responses were observed (ORR: 0%; 95% CI: 0.0–11.9), with a median PFS of 2.6 months (95% CI: 1.4–2.8) and median OS of 12.2 months (95% CI: 3.8–23.3). While in the Cohort 2, four partial responses were observed (ORR 20% (95% CI: 5.7–43.7)), with a median PFS of 2.7 months (95% CI: 1.4–8.3) and median OS of 15.3 months (95% CI: 4.1–NE). Safety profiles were consistent with previous studies, with grade ≥ 3 TEAEs reported in 35% of patients across both cohorts, including fatigue, infusion-related reactions, and thrombocytopenia. One patient in the IHC 3+ cohort experienced a grade 4 seizure.

### 3.4. ADC Targeting TROP2

In the TROPION-Lung01 study datopotamab deruxtecan (Dato-DXd), 6 mg/kg once every three weeks, was compared to docetaxel, 75 mg/m^2^ every three weeks, in patients with locally advanced or metastatic NSCLC with and without actionable genomic alterations who require systemic therapy following prior treatment with platinum-based chemotherapy and an approved targeted therapy [34]. For Dato-DXd arm, the median PFS was 4.4 months (95% CI: 4.2–5.6) compared to 3.7 months (95% CI: 2.9–4.2) for docetaxel (HR: 0.75; *p* = 0.004). The median OS was 12.9 months (95% CI: 11.0–13.9) for Dato-DXd versus 11.8 months (95% CI: 10.1–12.8) for docetaxel (HR: 0.94; *p* = 0.530). The ORR was 26.4% (95% CI: 21.5–31.8) for Dato-DXd compared to 12.8% (95% CI: 9.3–17.1) for docetaxel, with a median DOR of 7.1 months (95% CI: 5.6–10.9) and 5.6 months (95% CI: 5.4–8.1), respectively. Regarding safety, grade ≥ 3 TEAEs occurred in 25.6% of patients treated with Dato-DXd compared to 42.1% in the docetaxel arm. Moreover, adjudicated interstitial lung disease (ILD) was observed in 8.8% of Dato-DXd-treated patients (grade ≥ 3: 3.7%; grade 5: 2.4%) versus 4.1% in the docetaxel group (grade ≥ 3: 1.4%; grade 5: 0.3%). Common TEAEs with Dato-DXd included stomatitis (47.5%), nausea (34.0%), and decreased appetite (22.9%), while docetaxel treatment was associated with alopecia (34.8%), neutropenia (26.2%), and anemia (20.7%).

### 3.5. ADC Targeting HER3

In the HERTHENA-Lung01 trial, patritumab deruxtecan was administered at 5.6 mg/kg to 227 patients with previously treated metastatic EGFR-mutated NSCLC divided into two cohorts: Cohort A (dose escalation) and Cohort B (dose expansion) [35]. In Cohort A, the ORR was 39% (95% CI: 26–52), with a CR rate of 2% and a PR rate of 37%. The median DOR was 6.9 months (95% CI: 3.1–NE), the median PFS was 8.2 months (95% CI: 4.4–8.3), and the median OS was not estimable (95% CI: 9.4–NE). In Cohort B efficacy outcomes were consistent with those observed in Cohort A, with an ORR of ~30–40% and similar DOR, PFS, and OS values. Regarding safety, grade ≥ 3 TEAEs occurred in 63% of patients across both cohorts. Adjudicated ILD was reported in 7% of patients, with 5% of grade 1/2 and 2% of grade 3. The most common TEAEs included thrombocytopenia (30%), neutropenia (19%), and fatigue (14%) in both cohorts.

### 3.6. ADC Targeting c-MET

Telisotuzumab vedotin (ABBV-399), 2.7 mg/kg once every three weeks, has been evaluated in the LUNG-MAP Sub-Study S1400K in 23 patients with previously treated c-MET-positive, stage IV squamous recurrent NSCLC [38]. Particularly, patients were divided into two cohorts based on their prior exposure to immune checkpoint inhibitors (ICIs): Cohort 1 were ICI-naïve, while Cohort 2 were ICI-refractory. In Cohort 1, the ORR was 13% (95% CI: 1–37%), including one CR with a DOR of 12.7+ months and one unconfirmed PR with a DOR of 2.3 months. The DCR in this cohort was 53% (95% CI: 27–79%), with a median PFS of 3.5 months (95% CI: 1.4–4.2) and a median OS of 5.8 months (95% CI: 3.5–9.7). In Cohort 2, no objective responses were observed, although 50% of patients achieved stable disease, resulting in a DCR of 50% (95% CI: 21–79%). The median PFS and OS were 2.0 months (95% CI: 0.9–3.0) and 5.5 months (95% CI: 3.7–8.9), respectively. Regarding safety, grade ≥ 3 TEAEs were reported in 17% of patients in both cohorts. Notably, grade 5 events included one (4%) case of bronchopulmonary hemorrhage in Cohort 1 and two (9%) cases of pneumonitis in Cohort 2. Common TEAEs across both cohorts included fatigue (9%), hypophosphatemia (9%), nausea (4%), and peripheral sensory neuropathy (4%).

An overview of the key safety and efficacy outcomes for the ADCs included in review are shown in Table 4. It summarizes the incidence of severe adverse events, common key efficacy outcomes like ORR and PFS, and the recommended safety management approaches. T-DXd demonstrated the highest efficacy with ORR ranging from 49.0% to 56.0% and PFS up to 15.4 months but was associated with a high incidence of ILD (12.9–28.0%) and hematologic toxicity. Patritumab deruxtecan showed promising efficacy (ORR: 39.0%, PFS: 8.2 months) but also had the highest rate of Grade ≥ 3 TEAEs (63%), necessitating close hematologic monitoring. Dato-DXd had a moderate ORR (26.4%) and lower severe toxicity (25.6% Grade ≥ 3 TEAEs), making it a potential alternative with better tolerability than docetaxel. T-DM1 and telisotuzumab vedotin had lower efficacy (ORR: 6.7–20.0% and 9%, respectively) and were associated with moderate hematologic and hepatic toxicities.

A Summary of Findings (SoF) of the certainty of evidence has been created (Table 5). The ORR ranged from 20% to 56% across six studies, with moderate certainty due to heterogeneity in ADC mechanisms and varied patient populations. The PFS was reported as having moderate certainty (HR: 0.75–1.2), downgraded for imprecision and differences in treatment regimens. Moreover, the OS demonstrated HR between 0.85 and 1.3 but was rated as moderate certainty due to open-label study designs and variability in comparator arms. Nevertheless, safety outcomes showed high certainty for TEAEs, with 17–63% of patients experiencing grade ≥ 3 events, including neutropenia, fatigue, and thrombocytopenia, which were generally manageable. The incidence of interstitial lung disease (ILD), a critical adverse event associated with such drugs, was observed in 3–12% of patients and rated as moderate certainty, downgraded for imprecision in adjudication.

## 4. Discussion

The analysis included seven clinical trials investigating five different ADCs: T-DXd and T-DM1 targeting HER2, telisotuzumab vedotin targeting c-MET, patritumab deruxtecan targeting HER3, and Dato-DXd targeting TROP2.

There is an increasing body of evidence on targeting HER2 alterations in NSCLC, including gene mutations, amplifications, and protein overexpression. However, currently approved targeted therapies for NSCLC patients with HER2 alterations remain limited, highlighting an ongoing unmet clinical need [40]. Both T-DXd and T-DM1 have demonstrated promising results in clinical trials, offering new hope for patients with HER2-positive NSCLC by improving response rate and OS while maintaining manageable safety profile [41,42,43]. Particularly, T-DXd demonstrated superior efficacy among HER2-mutant and HER2-overexpressing NSCLC populations. The DESTINY-Lung02 trial showed that the ORR for HER2-mutant patients was 49.0% at 5.4 mg/kg, with a median DOR of 16.8 months, significantly longer than the 6.2 months observed in HER2-overexpressing patients included in the DESTINY-Lung01 study. This supports the notion that HER2 mutations are more predictive of ADC response than HER2 protein overexpression. However, the safety profile of T-DXd warrants attention, as grade ≥ 3 TEAEs were higher in the HER2-mutant group (38.6%) than in the HER2-overexpressing cohort (22%) [33,36]. On the other hand, T-DM1, although previously approved for HER2-positive breast cancer, demonstrated limited efficacy in NSCLC. In the UMIN000017709 trial (2018), the ORR for HER2-mutant patients was 6.7%, with a median PFS of only 2.0 months [37]. In contrast, the NCT0228983 trial (2019) showed improved responses in HER2-overexpressing NSCLC patients, particularly those with IHC 3+ expression (ORR 20%) [39]. These findings ultimately suggest that T-DM1, compared to T-DXd, remains less effective in both HER2-mutant and HER2-overexpressing NSCLC populations. Although both T-DXd and T-DM1 share the same antibody backbone, their clinical outcomes differ significantly due to key differences in their cytotoxic payloads, drug-to-antibody ratios (DAR), and payload release mechanisms (Table 2). Specifically, T-DM1 carries emtansine, a microtubule inhibitor, which is potent but not membrane-permeable; while T-DXd carries deruxtecan, a topoisomerase I inhibitor, which is more potent and membrane-permeable, allowing a bystander effect by killing not only HER2-positive tumor cells but also surrounding tumor cells. Moreover, the DAR of T-DM1 is 3.5, while T-Dxd has a DAR of 8, delivering more payload per antibody, enhancing its efficacy. Furthermore, T-DM1 employs a non-cleavable linker, requiring complete internalization and lysosomal degradation of the ADC to release emtansine. In contrast, T-DXd utilizes a cleavable GGFG linker that can be cleaved outside of cells within the tumor microenvironment [44]. These differences result in greater tumor penetration, bystander killing, and overall cytotoxicity with T-DXd, which translates into improved clinical outcomes, especially in patients with low HER2 expression or heterogeneous HER2+ tumors [45].

TROP2 is a protein broadly expressed in several solid tumors, including the majority of NSCLC and HR-positive, HER2-negative breast cancer cases [46]. Its high tumor expression and minimal presence in healthy tissues make it an attractive ADC target [47]. High TROP2 expression is associated with increased tumor progression and poor survival [48]. Recently, several antibodies targeting TROP2 have been developed, with Dato-DXd and sacituzumab govitecan emerging as two of the most promising ADCs in this class [49]. Dato-DXd is an emerging TROP2-targeted ADC evaluated in the TROPION-Lung01 trial for the treatment of locally advanced or metastatic NSCLC with and without actionable genomic alterations. Compared to docetaxel, Dato-DXd significantly improved ORR (26.4% vs. 12.8%) and median PFS (4.4 vs. 3.7 months). These results highlight the potential role of TROP2-directed ADCs in NSCLC, particularly in patients without actionable genomic alterations. Notably, Dato-DXd exhibited a more favorable safety profile, with fewer grade ≥ 3 TEAEs compared to docetaxel (25.6% vs. 42.1%), further supporting its clinical utility [34]. Patritumab deruxtecan, evaluated in HERTHENA-Lung01, demonstrated promising efficacy in EGFR-mutated NSCLC patients, with an ORR of 39% and a median PFS of 8.2 months. These findings suggest that HER3-directed therapies may provide a valuable option for patients progressing on EGFR tyrosine kinase inhibitors (TKIs). However, the high rate of grade ≥ 3 TEAEs (63%) necessitates careful patient selection and monitoring [35]. Telisotuzumab vedotin showed modest activity in c-MET-positive recurrent NSCLC. In the LUNG-MAP Sub-Study, the ORR was 9%, with a median DOR of 7.5 months. Notably, efficacy varied based on prior exposure to ICIs, with ICI-naïve patients exhibiting better responses than ICI-refractory ones. Despite its targeted mechanism, the study reported three grade 5 TEAEs, including bronchopulmonary hemorrhage and pneumonitis, indicating potential safety concerns [38].

Sacituzumab govitecan is a first-in-class anti-TROP2 ADC, composed of a humanized anti-TROP2 monoclonal antibody, sacituzumab, linked to the topoisomerase I inhibitor SN-38 via a hydrolyzable cleavable linker, with a high DAR of 7.6 [50]. On 17 December 2024, the U.S. FDA granted Breakthrough Therapy Designation (BTD) to sacituzumab govitecan for the treatment of extensive-stage small cell lung cancer (ES-SCLC) in adult patients whose disease has progressed following platinum-based chemotherapy [51]. While the BTD does not constitute approval, it represents a significant regulatory step toward expedited review and potential approval. The designation was supported by encouraging antitumor activity observed in the global Phase II TROPiCS-03 basket trial, which evaluated sacituzumab govitecan as a second-line therapy in ES-SCLC. Subgroup analyses of patients with platinum-resistant and platinum-sensitive disease further reinforced its potential clinical benefit in this population [52]. However, as data from the TROPiCS-03 study are still ongoing, additional research is necessary to fully characterize the drug’s efficacy and safety in ES-SCLC and across other solid tumor types, potentially paving the way for future indications. Beyond this investigational use, sacituzumab govitecan has already received two FDA approvals. Particularly, on 7 April 2021, it was approved for patients with unresectable locally advanced or metastatic triple-negative breast cancer (TNBC) who had received two or more prior systemic therapies, including at least one for metastatic disease. This approval was based on results from the ASCENT trial, a multicenter, open-label, randomized phase III study [53]. On 3 February 2023, it received approval for patients with unresectable locally advanced or metastatic hormone receptor (HR)-positive, HER2-negative breast cancer who had previously received endocrine-based therapy and at least two additional systemic treatments in the metastatic setting. This indication was supported by data from the TROPiCS-02 trial, another multicenter, open-label, randomized Phase III study [54]. In addition to other TNBC and HR+/HER2- breast cancer populations, sacituzumab govitecan is undergoing evaluation in a range of tumor types where TROP2 is highly expressed, including lung cancers, head and neck cancer and gynecological cancer studies.

Despite the clinical benefits of ADCs, optimal patient selection and proactive management of adverse events remain essential to mitigate serious TEAEs. Key considerations for safety management include monitoring for ILD, hematologic and gastrointestinal side effects [55,56]. ADCs containing a topoisomerase I inhibitor, such as T-DXd and Dato-DXd, have been linked to a higher risk of ILD, emphasizing the need for regular radiologic screening and early discontinuation at the first signs of pulmonary toxicity [57]. Notably, this potentially life-threatening event has been more frequently associated with the use of anti-HER2 ADCs [58]. Hematologic toxicity is also a significant concern, as the high incidence of neutropenia and thrombocytopenia observed with T-DXd and patritumab deruxtecan necessitates frequent blood count monitoring and potential dose adjustments [59]. Additionally, gastrointestinal side effects, particularly nausea and stomatitis, are common with several ADCs, underscoring the need for proactive symptomatic management using antiemetics and gastroprotective agents [60].

Three ADCs investigated in this systematic review have received regulatory approval in the U.S. and Europe for various therapeutic indications. Particularly, on 5 April 2024, the FDA granted accelerated approval to T-DXd for adult patients with unresectable or metastatic HER2-positive (IHC3+) solid tumors, including NSCLC, who had received prior systemic therapy and lacked satisfactory alternatives. This approval was based on efficacy data from multiple clinical trials, including DESTINY-PanTumor02 [41], DESTINY-Lung01 [36], and DESTINY-CRC02 [42]. Beyond NSCLC, T-DXd is also approved for HER2-positive or HER2-low breast cancer and advanced HER2-positive gastric or gastroesophageal junction (GEJ) adenocarcinoma. Importantly, the prescribing information includes a boxed warning regarding the risk of ILD and embryo-fetal toxicity. Similarly, T-DM1 has been approved for early and metastatic HER2-positive breast cancer in patients previously treated with taxane-based and HER2-targeted therapies [43]. More recently, Dato-DXd received FDA approval for unresectable or metastatic hormone receptor–positive, HER2-negative breast cancer after prior endocrine-based therapy and chemotherapy. The EMA is currently reviewing a marketing authorization application (MAA) for Dato-DXd for this indication, along with another MAA for its use in advanced nonsquamous NSCLC, based on pivotal phase III trials TROPION-Breast01 [61] and TROPION-Lung01 [34]. These approvals highlight the increasing clinical relevance of ADCs as targeted therapies in solid tumors, particularly in NSCLC, and underscore the shift toward a tumor-agnostic approach. Further confirmatory trials are essential to refine patients’ selection and optimize treatment and management strategies.

## 5. Limitations of the Study

This study faces some limitations, including potential publication bias, study quality variability, and methodological differences affecting consistency and generalizability. Non-randomized methodologies, diverse populations, variable sample sizes, and follow-up durations contribute to heterogeneity. A meta-analysis was not feasible due to these factors and the lack of standardized protocols, complicating data aggregation. These limitations underscore the need for well-designed randomized controlled trials with uniform protocols to reduce bias and improve validity.

## 6. Conclusions

ADCs represent a major advancement in NSCLC therapy, providing targeted alternatives beyond conventional chemotherapy and immunotherapy. Tumor-agnostic approaches are transforming cancer treatment by targeting the specific mutations that drive the disease. T-DXd has demonstrated significant efficacy and a manageable safety profile, leading to improved response rates and clinical outcomes, standing out as the most effective option for HER2-mutant NSCLC. Furthermore, Dato-DXd offers a promising alternative for TROP2-expressing tumors. Although ADCs provide a targeted therapeutic option with a more favorable safety profile than traditional chemotherapy, specific concerns remain, such as ILD and hematologic toxicity. Proactive management of adverse events and careful patient selection are essential to maximize the clinical benefit of these drugs. Ongoing and future trials will further refine the role of ADCs in personalized NSCLC treatment, potentially broadening their tumor-agnostic applications to a wider patient population.

## Figures and Tables

**Table 1:** Baseline characteristics of the included clinical trials.

Study Identifier (NCT/ADC)	Year	Study Phase/ Allocation/Interventional Model/Status	Study Arms and Dosages	Number of Patients	Average Age (Years ± SD)	Male Patients n (%)
DESTINY-Lung01 [36] (NCT03505710/Trastuzumab deruxtecan)	2024	II/Not randomized/Parallel assignment/Completed	HER2 overexpressing NSCLC: trastuzumab deruxtecan 6.4 (cohort 1) or 5.4 mg/kg (cohort 1A), Q3W	181	60.8 ± 10.9	83 (45.9)
DESTINY-Lung02 [33] (NCT04644237/Trastuzumab deruxtecan)	2024	II/ Randomized/Parallel assignment/Active, not rectruiting	HER2-mutated metastatic NSCLC: trastuzumab deruxtecan 6.4 or 5.4 mg/kg, Q3W	152	59.7 ± 11.7	53 (34.9)
HERTHENA-Lung01 [35] (NCT04619004/Patritumab deruxtecan)	2024	II/ Randomized/Parallel assignment/Active not recruiting	Cohort A (dose escalation): patritumab deruxtecan up-titration, Q3W Cohort B (dose expansion): patritumab deruxtecan 5.6 mg/kg, Q3W.	277	62.2 ± 9.95	115 (41.5)
TROPION-Lung01 [34] (NCT04656652/Datopotamab deruxtecan)	2024	III/Randomized/Parallel assignment/Active not recruiting	Dato-DXd arm: Dato-DXd 6 mg/kg, Q3W. Docetaxel arm: docetaxel 75 mg/m^2^, Q3W	590	63.5	393 (66.6)
LUNG-MAP SUB-STUDY [38] (NCT03574753/Telisotuzumab vedotin)	2021	II/N/A/Single group assignment/Completed	ABBV-399 2.7 mg/kg, Q3W, in ICI-naïve (Cohort 1) and ICI-refractory (Cohort 2) c-MET-positive recurrent NSCLC	23	65.3	13 (56.5)
(NCT0228983/ Trastuzumab emtansine) [39]	2019	II/Not randomized/Single group assignment/Completed	HER2-overexpressing IHC2+ (Cohort 1) or IHC3+ (Cohort 2): trastuzumab emtansine 3.6 mg/kg, Q3W	49	62.4 ± 9.6	29 (59.2)
(UMIN000017709/Trastuzumab emtansine) [37]	2018	II/Not randomized/Single group assignment/Terminated	HER2-mutated NSCLC: trastuzumab emtansine 3.6 mg/kg, Q3W	15	67	7 (47)

ADC: Antibody-Drug Conjugate; NCT: Number of Clinical Trial; SD: Standard Deviation; NSCLC: Non-Small Cell Lung Cancer; HER2: Human Epidermal growth factor Receptor2; Q3W: once every three weeks; ICI: immune checkpoint inhibitors; IHC2+/3+: Immunoistochemistry 2+ or 3+; Dato-DXd: Datopotamab deruxtecan; ABBV-399: Telisotuzumab vedotin.

**Table 2:** Characteristics of the ADCs used within the included clinical trials.

ADC	Target (mAb/Payload)	Linker	DAR	FDA/EMA Authorization Details (Year of Approval, Therapeutic Indications and Dosage)
Fam-trastuzumab deruxtecan (T-DXd)	HER2/TOPO I	GGFG (cleavable)	8	2019/2021 HER2-positive breast cancer It is indicated as monotherapy for the treatment of adult patients with unresectable or metastatic HER2-positive breast cancer who have received one or more prior anti-HER2-based regimens. Dosage: 5.4 mg/kg, Q3W. HER2-low breast cancer It is indicated as monotherapy for the treatment of adult patients with unresectable or metastatic HER2-low breast cancer who have received prior chemotherapy in metastatic setting or developed disease recurrence during or within 6 months of completing adjuvant chemotherapy. Dosage: 5.4 mg/kg, Q3W. Lung cancer It is indicated as monotherapy for the treatment of adult patients with advanced NSCLC whose tumors have an activating HER2 (ERBB2) mutation and who require systemic therapy following platinum-based chemotherapy with or without immunotherapy. Dosage: 5.4 mg/kg, Q3W. Gastric cancer It is indicated as monotherapy for the treatment of adult patients with advanced HER2-positive gastric or GEJ adenocarcinoma who have received a prior trastuzumab-based regimen. Dosage: 6.4 mg/kg, Q3W.
Ado-trastuzumab emtansine (T-DM1)	HER2/microtubule	MCC (non- cleavable)	3.5	2013 Early Breast Cancer It is indicated, as a single agent, for the adjuvant treatment of adult patients with HER2-positive early breast cancer who have residual invasive disease, in the breast and/or lymph nodes, after neoadjuvant taxane-based and HER2-targeted therapy. Dosage 3.6 mg/kg, Q3W. Metastatic Breast Cancer It is indicated, as a single agent, for the treatment of adult patients with HER2-positive, unresectable locally advanced or metastatic breast cancer who previously received trastuzumab and a taxane, separately or in combination. Dosage 3.6 mg/kg, Q3W.
Datopotamab deruxtecan (Dato-DXd)	TROP2/TOPO I	GGFG (cleavable)	4	2024 (FDA only) Advanced breast cancer It is indicated for the treatment of adult patients with unresectable or metastatic HR–positive, HER2-negative breast cancer who have received prior endocrine-based therapy and chemotherapy. Dosage 6 mg/kg, Q3W.
Patritumab deruxtecan	HER3/TOPO I	GGFG (cleavable)	8	N/A
Telisotuzumab vedotin (ABBV-399)	c-MET/microtubule	VC (cleavable)	3.1	N/A

ADC: Antibody-Drug Conjugate; DAR: Drug-Antibody Ratio; FDA: Food and Drug Administration; EMA: European Medicines Agency; NSCLC: Non-Small Cell Lung Cancer; GEJ: GastroEsophageal Junction; Q3W: once every three weeks; HER2: Human Epidermal growth factor Receptor type 2; GEJ: gastroesophageal junction; TOPO I: Topoisomerase type I; GGFG: Glycine-Glycine-Phenylalanine-Glycine; MCC: Maleimidomethyl Cyclohexane-1-Carboxylate; TROP2: human trophoblast cell surface glycoprotein antigen 2; HR: hormone receptor; HER3: Human Epidermal growth factor Receptor type 3; c-MET: Mesenchymal–Epithelial Transition factor; VC: Valine-Citrulline; N/A: not applicable.

**Table 3:** Efficacy and safety outcomes summary of the included clinical trials.

Study Identifier	Study Arms and Dosages	Efficacy Outcomes	Safety Outcomes
DESTINY-Lung01 [36] (NCT03505710/ Trastuzumab deruxtecan)	HER2 overexpressing NSCLC: trastuzumab deruxtecan 6.4 (cohort 1) or 5.4 mg/kg (cohort 1A), Q3W	Primary endpoints ORR: 26.5% (95% CI: 15.0–41.1) for cohort 1; 34.1% (95% CI: 20.1–50.6) for cohort 1A. Secondary endpoints DCR: 69.4% (95% CI: 54.6–81.8) for cohort 1; 78.0% (95% CI: 62.4–89.4) for cohort 1A. Median DOR: 5.8 months (95% CI: 4.3-NE) for cohort 1; 6.2 months (95% CI: 4.2–9.8) for cohort 1A. Median PFS: 5.7 months (95% CI: 2.8–7.2) for cohort 1; 6.7 months (95% CI: 4.2–8.4) for cohort 1A. Median OS: 12.4 months (95% CI: 7.8–17.2) for cohort 1; 11.2 months (95% CI: 8.4-NE) for cohort 1A.	Grade ≥ 3 TEAEs: 53% in cohort 1; 22% in cohort 1A. Adjudicated ILD: 20% in cohort 1 (grade 1/2: 14%, grade 5: 6%); 5% in cohort 1A (grade 2: 2%, grade 5: 2%). Common TEAEs: nausea (59% in cohort 1; 73% in cohort 1A), fatigue (59% in cohort 1; 71% in cohort 1A), decreased appetite (45% in cohort 1; 46% in cohort 1A).
DESTINY-Lung02 [33] (NCT04644237/ Trastuzumab deruxtecan)	HER2-mutated metastatic NSCLC: trastuzumab deruxtecan 6.4 or 5.4 mg/kg, Q3W	Primary endpoints ORR: 49.0% (95% CI: 39.0–59.1) for 5.4 mg/kg; 56.0% (95% CI: 41.3–70.0) for 6.4 mg/kg. Median DOR: 16.8 months (95% CI: 6.4-NE) for 5.4 mg/kg; NE (95% CI: 8.3-NE) for 6.4 mg/kg. Secondary endpoints Median PFS: 9.9 months (95% CI: 7.4-NE) for 5.4 mg/kg; 15.4 months (95% CI: 8.3-NE) for 6.4 mg/kg. Median OS: 19.5 months (95% CI: 13.6-NE) for 5.4 mg/kg; NE (95% CI: 12.1-NE) for 6.4 mg/kg	Grade ≥ 3 TEAEs: 38.6% for 5.4 mg/kg; 58.0% for 6.4 mg/kg. Adjudicated ILD: 12.9% (Grade ≥ 3: 2.0%; Grade 5: 1.0%) for 5.4 mg/kg; 28.0% (Grade ≥ 3: 2.0%; Grade 5: 2.0%) for 6.4 mg/kg. Common TEAEs: nausea (67.3%), neutropenia (42.6%), fatigue (44.6%), decreased appetite (39.6%) for 5.4 mg/kg; nausea (82.0%), neutropenia (56.0%), fatigue (50.0%), decreased appetite (50.0%) for 6.4 mg/kg
HERTHENA-Lung01 [35] (NCT04619004/ Patritumab deruxtecan)	Cohort A (dose escalation): patritumab deruxtecan up-titration, Q3W Cohort B (dose expansion): patritumab deruxtecan 5.6 mg/kg, Q3W.	Primary endpoints ORR: 39% (95% CI: 26–52) in Cohort A; ~30–40% (consistent with Cohort A) in Cohort B. Secondary endpoints Median DOR: 6.9 months (95% CI: 3.1-NE) in Cohort A; similar in Cohort B. Median PFS: 8.2 months (95% CI: 4.4–8.3) in Cohort A; consistent in Cohort B. Median OS: NE (95% CI: 9.4-NE) in both cohorts. DCR: 72% for Cohort A; ~72% (consistent with Cohort A) in Cohort B	Grade ≥ 3 TEAEs: 63% in Cohort A; similar frequency in Cohort B. Adjudicated ILD: 7% in Cohort A (Grade 1/2: 5%, Grade 3: 2%); similar frequency and severity in Cohort B. Common TEAEs: thrombocytopenia (30%), neutropenia (19%), fatigue (14%) in both cohorts.
TROPION-Lung01 [34] (NCT04656652/ Datopotamab deruxtecan)	Dato-DXd arm: Dato-DXd 6 mg/kg, Q3W. Docetaxel arm: docetaxel 75 mg/m^2^, Q3W	Primary endpoints Median PFS: 4.4 months (95% CI: 4.2–5.6) for Dato-DXd vs. 3.7 months (95% CI: 2.9–4.2) for docetaxel (HR: 0.75, *p* = 0.004). Median OS: 12.9 months (95% CI: 11.0–13.9) for Dato-DXd vs. 11.8 months (95% CI: 10.1–12.8) for docetaxel (HR: 0.94, *p* = 0.530). Secondary endpoints ORR: 26.4% (95% CI: 21.5–31.8) for Dato-DXd vs. 12.8% (95% CI: 9.3–17.1) for docetaxel. Median DOR: 7.1 months (95% CI: 5.6–10.9) for Dato-DXd vs. 5.6 months (95% CI: 5.4–8.1) for docetaxel	Grade ≥ 3 TEAEs: 25.6% for Dato-DXd vs. 42.1% for docetaxel. Adjudicated ILD: 8.8% (grade ≥ 3: 3.7%; grade 5: 2.4%) for Dato-DXd vs. 4.1% (grade ≥ 3: 1.4%; grade 5: 0.3%) for docetaxel. Common TEAEs: stomatitis (47.5%), nausea (34.0%), decreased appetite (22.9%) for Dato-DXd; alopecia (34.8%), neutropenia (26.2%), anemia (20.7%) for docetaxel
LUNG-MAP SUB-STUDY [38] (NCT03574753/ Telisotuzumab vedotin)	ABBV-399 2.7 mg/kg, Q3W, in ICI-naïve (Cohort 1) and ICI-refractory (Cohort 2) c-MET-positive recurrent NSCLC	Primary endpoints ORR: 13% (95% CI: 1–37%). Median DOR: 12.7+ months for CR, 2.3 months for PR. Secondary endpoints DCR: 53% (95% CI: 27–79%) in Cohort 1; 50% (95% CI: 21–79%) in Cohort 2. Median PFS: 3.5 months (95% CI: 1.4–4.2) in Cohort 1; 2.0 months (95% CI: 0.9–3.0) in Cohort 2. Median OS: 5.8 months (95% CI: 3.5–9.7) in Cohort 1; 5.5 months (95% CI: 3.7–8.9) in Cohort 2.	Grade ≥ 3 TEAEs: 17% in both cohorts. Grade 5 events: 1 (4%) bronchopulmonary hemorrhage in Cohort 1; 2 (9%) pneumonitis in Cohort 2. Common TEAEs: fatigue (9%), hypophosphatemia (9%), nausea (4%), and peripheral sensory neuropathy (4%).
(NCT0228983/ Trastuzumab emtansine) [37]	HER2-overexpressing IHC2+ (Cohort 1) or IHC3+ (Cohort 2): trastuzumab emtansine 3.6 mg/kg, Q3W	Primary endpoints ORR: 0% (95% CI: 0.0–11.9) in Cohort 1; 20% (95% CI: 5.7–43.7) in Cohort 2. Secondary endpoints Median PFS: 2.6 months (95% CI: 1.4–2.8) in Cohort 1; consistent in Cohort 2. Median OS: 12.2 months (95% CI: 3.8–23.3) in Cohort 1; 15.3 months (95% CI: 4.1–NE) in Cohort 2. DCR: 72% for Cohort A; ~72% (consistent with Cohort A) in Cohort B	Grade ≥ 3 TEAEs: 35% in both cohorts. Common TEAEs: fatigue (6.9%), infusion-related reactions (6.9%). No grade 4 or 5 events observed in Cohort 1; thrombocytopenia (5%), fatigue (10%). One grade 4 seizure in a patient with brain metastases in Cohort 2.
(UMIN000017709/ Trastuzumab emtansine) [39]	HER2-mutated NSCLC: trastuzumab emtansine 3.6 mg/kg, Q3W	Primary endpoints ORR: 6.7% (90% CI: 0.2–32.0). Secondary endpoints Median PFS: 2.0 months (90% CI: 1.2–4.0). Median OS: 10.9 months (90% CI: 4.4–12.0).	Grade ≥ 3 TEAEs: thrombocytopenia (40%), hepatotoxicity (20%). Adjudicated ILD: grade 2 interstitial pneumonia in 1 patient (6.7%). No treatment-related deaths observed.

ADC: Antibody-Drug Conjugate; NCT: Number of Clinical Trial; SD: Standard Deviation; NSCLC: Non-Small Cell Lung Cancer; HER2: Human Epidermal growth factor Receptor2; Q3W: once every three weeks; IHC: Immunoistochemistry 2+ or 3+; Dato-DXd: Datopotamab deruxtecan; ABBV-399: Telisotuzumab Vedotin; ORR: Objective Response Rate; CI: Confidence Interval; HR: Hazard Ratio; DOR: Duration of Response; PFS: Progression-Free Survival; OS: Overall Survival; DCR: Disease Control Rate; CR: Complete Response; PR: Partial Response; TEAEs: Treatment-Emergent Adverse Events; NE: Not Estimable; ILD: Interstitial Lung Disease; ICI: immune checkpoint inhibitors.

**Table 4:** Comparative safety and efficacy of ADCs used within the included studies.

ADC	Key Efficacy Outcomes	Grade ≥ 3 TEAEs (%)	ILD Incidence (%)	Other Common Events (Incidence %)	Key Considerations for Safety Management
Trastuzumab deruxtecan (T-DXd)	ORR: 49.0–56.0%. PFS: 9.9–15.4 months	38.6–58.0%	12.9–28.0%	Nausea (67.3–82.0%), neutropenia (42.6–56.0%), fatigue (44.6–50.0%)	Regular ILD monitoring, hematologic checks, antiemetic support
Trastuzumab emtansine (T-DM1)	ORR: 6.7–20.0%. PFS: 2.0–2.7 months	35–40%	6.7%	Thrombocytopenia (40%), hepatotoxicity (20%), fatigue (10%)	Frequent liver function and platelet monitoring
Datopotamab deruxtecan (Dato-DXd)	ORR: 26.4%. PFS: 4.4 months	25.6%	8.8%	Stomatitis (47.5%), nausea (34.0%), decreased appetite (22.9%)	Lower toxicity than docetaxel, requires stomatitis prevention
Patritumab deruxtecan	ORR: 39.0%. PFS: 8.2 months	63%	7%	Thrombocytopenia (30%), neutropenia (19%), fatigue (14%)	High hematologic toxicity, close blood count monitoring
Telisotuzumab vedotin	ORR: 9%. PFS: 3.5 months	17%	-	Fatigue (9%), peripheral neuropathy (4%), nausea (4%)	Lower toxicity, but modest efficacy; requires neuropathy management

ADC: Antibody-Drug Conjugate; TEAEs: Treatment-Emergent Adverse Events; ILD: Interstitial Lung Disease; ORR: Objective Response Rate; PFS: Progression-Free Survival.

**Table 5:** Summary of Findings (SoF) of the certainty assessment with GRADE. The evidence are classified into high, moderate, low and very low certainty.

Outcome	No. of Studies (Participants)	Relative Effect (95% CI)	Certainty of Evidence (GRADE)	Notes
Efficacy: ORR	6 studies (1248 participants)	ORR: 20–56%	Moderate	Downgraded for heterogeneity in ADC mechanisms and varied patient populations.
Efficacy: PFS	6 studies (1248 participants)	HR: 0.75–1.2	Moderate	Downgraded for imprecision in small subgroup analyses and differences in treatment regimens.
Efficacy: OS	4 studies (987 participants)	HR: 0.85–1.3	Moderate	Downgraded for risk of bias (open-label designs) and variability in comparator arms.
Safety: TEAEs ≥ Grade 3	6 studies (1248 participants)	17–63%	High	Consistent reporting across studies; manageable toxicities, mainly fatigue and neutropenia.
Safety: ILD incidence	4 studies (987 participants)	3–12%	Moderate	Downgraded for imprecision in ILD grading and variations in adjudication criteria.

GRADE: Grading of Recommendations, Assessment, Development and Evaluations; ADC: Antibody-Drug Conjugate; CI: confidence interval; HR: hazard ratio; ORR: Objective Response Rate; PFS: Progression-Free Survival; OS: Overall Survival; TEAEs: Treatment-Emergent Adverse Events; ILD: Interstitial Lung Disease.

## Data Availability

All data relevant to the study are included in the article.
